# Associations of Hand-Grip Strength and Arm Muscle Mass Index Relative to Body Weight with Risk of Non-Alcoholic Fatty Liver Disease in Wheelchair Users

**DOI:** 10.3390/nu18111715

**Published:** 2026-05-27

**Authors:** Minjun Kim, Jeonghyeon Kim, Inhwan Lee

**Affiliations:** 1 Research Institute of Sports Science, Changwon National University, Changwon 51140, Republic of Korea; kyj1103_2@naver.com; 2 Department of Physical Education, Changwon National University, Changwon 51140, Republic of Korea; spep08@cwnu.ac.kr

**Keywords:** hand-grip strength, arm muscle mass, non-alcoholic fatty liver disease, wheelchair users, person with disability

## Abstract

**Background**: This study examined the independent and combined associations of hand-grip strength (HGS) and arm muscle mass index with risk of non-alcoholic fatty liver disease (NAFLD) in individuals who use wheelchairs. **Methods**: Eighty-five individuals aged ≥ 30 years who use wheelchairs were enrolled as volunteers from sports welfare facilities for individuals with disabilities in “G” and “C” provinces. HGS was measured using a dynamometer, whereas the arm muscle mass index was assessed using a body composition analyzer. Risk of NAFLD was defined as a hepatic steatosis index (HSI) > 36 and Framingham steatosis index (FSI) ≥ 23. **Results**: The low-HGS group (HSI, odds ratio [OR] = 3.589, *p* = 0.013; FSI, OR = 2.710, *p* = 0.031) had a significantly higher NAFLD risk compared to the high-HGS group (reference, OR = 1). The low-arm-muscle-mass-index group showed a higher risk of NAFLD (HSI, OR = 2.762, *p* = 0.043; FSI, OR = 3.493, *p* = 0.007) compared to the high-arm-muscle-mass-index group (reference, OR = 1). Combining the low-HGS + low-arm-muscle-mass-index group (HSI, OR = 6.849, *p* = 0.006; FSI, OR = 6.957, *p* = 0.002) showed a significantly higher risk of NAFLD compared to the high-HGS + high-arm-muscle-mass-index group (reference, OR = 1), even after adjusting for covariates (HSI, OR = 7.352, *p* = 0.006; FSI, OR = 7.406, *p* = 0.003). **Conclusions**: Low HGS and low arm muscle mass index are independently associated with a higher risk of NAFLD in individuals who use wheelchairs, with low HGS plus low arm muscle mass index further increasing this risk.

## 1. Introduction

Non-alcoholic fatty liver disease (NAFLD) refers to a spectrum of liver conditions characterized by excessive fat accumulation in the liver in the absence of significant alcohol consumption, drug use, or viral infection [1]. The disease progresses through various stages, ranging from steatosis, which typically presents with no discernible symptoms, to non-alcoholic steatohepatitis, which is characterized by inflammation and liver fibrosis. If left untreated, NAFLD can progress to severe conditions such as cirrhosis and liver cancer. Over the past decade, the global prevalence of NAFLD has increased rapidly, affecting approximately 25% of the general population [2]. Recognized as a significant public health concern, the increasing prevalence of NAFLD is clinically important because of its potential progression to more severe disease and its association with higher mortality from conditions such as cardiovascular disease and cancer [3,4,5].

Individuals with impaired mobility, such as those with spinal cord injury (SCI), brain lesions, or polio-related disabilities, often rely on wheelchairs as their primary mode of transportation. These individuals are more vulnerable to obesity, metabolic syndrome, and type 2 diabetes, all of which are major NAFLD risk factors because of limited opportunities for physical activity, sedentary lifestyles, and reduced energy expenditure from underutilized leg muscles [6,7,8,9]. Consequently, they may be at greater risk of NAFLD than individuals without disabilities. Indeed, Eisenberg et al. [10] found that individuals with chronic SCI had a significantly higher incidence of NAFLD than individuals without SCI. Moreover, epidemiological studies have reported that NAFLD prevalence among individuals with SCI ranges from approximately 49 to 60% [11,12]. Despite this high prevalence, research on NAFLD prevention and treatment among individuals who use wheelchairs remains scarce, which limits efforts to protect and promote their health from a broader public health perspective.

Muscle loss resulting from physical inactivity and imbalanced nutrition can promote insulin resistance, chronic inflammation, and decreased beneficial myokine secretion, thereby contributing to NAFLD onset and progression [13]. Epidemiological studies have linked NAFLD to decreased skeletal muscle mass and low hand-grip strength (HGS) [14,15], and concurrent losses of muscle mass and strength may further increase NAFLD risk [16]. These findings underscore the potential usefulness of muscle mass and strength as screening tools for identifying NAFLD risk, with combined assessments offering enhanced discriminatory power. However, these studies have mainly included individuals without disabilities, leaving the role of muscle mass and strength in NAFLD risk among individuals with disabilities insufficiently understood.

Individuals who use wheelchairs commonly experience muscle atrophy due to prolonged leg muscle inactivity and rely predominantly on upper body strength for mobility and daily activities [17]. Given the impact of physical activity on muscle health and the known associations of muscle mass and strength with risk of NAFLD in individuals without disabilities, the arm muscle mass and strength of individuals who use wheelchairs may be important factors associated with NAFLD risk. Thus, this study aimed to investigate the associations between muscle strength, arm muscle mass index (AMI), and risk of NAFLD among individuals who use wheelchairs.

## 2. Materials and Methods

### 2.1. Study Participants

**Figure 1:**
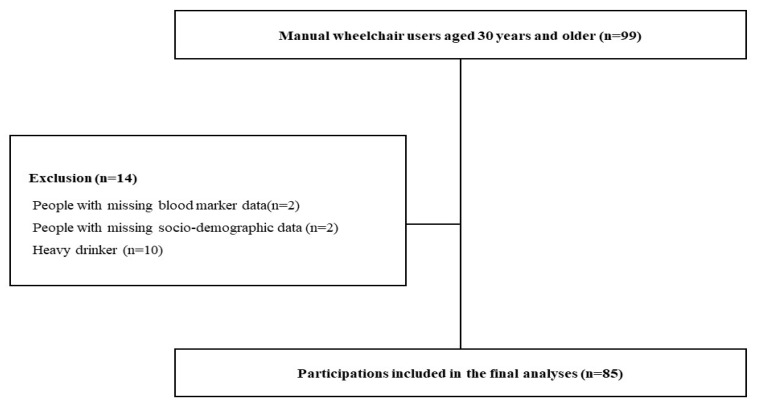
Flowchart of eligible participants in this study.

The present study was conducted between September 2022 and March 2023. The initial target population comprised 99 individuals aged ≥ 30 years who use wheelchairs and use sports welfare facilities for individuals with disabilities in the “G” and “C” provinces in Korea. Subsequently, 14 candidates were excluded due to missing marker data (*n* = 2), missing demographic questionnaire survey data (*n* = 2), and heavy alcohol consumption (≥15 servings per week for men and ≥8 servings per week for women; *n* = 10). Therefore, data from 85 participants were included in the final analysis (Figure 1). Individuals who were fully informed about the objectives, methods, and procedures of this study and voluntarily signed a consent form prior to the start of this study were eligible for participation. The present study was approved by the Institutional Review Board of Changwon National University (7001066-202401-HR-009).

### 2.2. Assessment of Body Composition and AMI

Body mass index (BMI), percent body fat, waist circumference (WC), arm muscle mass, and AMI were measured as body composition variables. BMI was calculated using the formula body weight (kg)/height (m^2^) based on height measured using an anthropometric tape measure while lying down, and body weight was measured using a wheelchair scale (AD-5105NP, AND, Bucheon, Republic of Korea) while the participant wore light clothing. WC was measured from the lowest rib to the midpoint of the iliac crest using an anthropometric tape measure with the participant in a seated position. Percent body fat and arm muscle mass were measured using a body composition analyzer (S10, Inbody, Seoul, Korea) based on bioelectrical impedance analysis. AMI was calculated using the following formula: arm muscle mass (kg)/body weight (kg) × 100 [18].

### 2.3. Blood Variables

Fasting blood glucose (FBG), high-density lipoprotein cholesterol (HDL-C), triglycerides (TG), aspartate aminotransferase (AST), alanine aminotransferase (ALT), and gamma-glutamyl transferase (GGT) were assessed as blood variables. For blood analysis, a 70 μL blood sample was collected from the tip of the index finger of the left hand after participants had fasted for at least 8 h, and the collected blood sample was immediately placed in a cartridge and analyzed using a dry chemistry 8 analyzer (Labgeo PT10, Samsung Electronics, Seoul, Republic of Korea).

### 2.4. Hand-Grip Strength

To assess HGS, a digital dynamometer (TKK-5401, Takei, Tokyo, Japan) was used to measure HGS twice in each hand, and the measured values were recorded in kg. The maximum measured value was considered the absolute HGS, while the relative HGS was calculated using the following formula: absolute HGS (kg)/body weight (kg) × 100. The calculated relative HGS values were categorized into the top and bottom 50% according to age and sex. The specific median cut-off values used for this categorization were as follows: for men, 55.3 (<60 years) and 45.9 (≥60 years); for women, 36.6 (<60 years) and 41.2 (≥60 years).

### 2.5. Definition of NAFLD (Hepatic Steatosis) Risk

Risk of NAFLD was defined on the basis of hepatic steatosis, and screening for hepatic steatosis was performed using the hepatic steatosis index (HSI) [19] and Framingham steatosis index (FSI) [20], both of which have demonstrated reliability and validity for the Korean population. The formulas used in the present study to calculate HSI and FSI and the cut-off points for screening the high-risk steatosis group were as follows:

HSI = 8 × (ALT/AST ratio) + BMI (+2, if diabetes mellitus; +2, if female)

A calculated score of >36 points was considered to indicate a higher risk of NAFLD:

FSI = e^x^/(1 + e^x^),

where X = −7.981 + 0.011 × age (years) − 0.146 × sex (female = 1, male = 0) + 0.173 × BMI (kg/m^2^) + 0.007 × TG (mg/dL) + 0.593 × hypertension (yes = 1, no = 0) + 0.789 × diabetes (yes = 1, no = 0) + 1.1 × ALT/AST ratio ≥ 1.33 (yes = 1, no = 0).

A calculated FSI score of ≥23 points was considered to indicate a higher risk of NAFLD.

### 2.6. Covariates

Socioeconomic status and health-related factors were assessed as covariates using the questionnaire from the Korean Panel Survey on Living with Disabilities [21]. Socioeconomic factors included average monthly household income, which was calculated by dividing the previous year’s income by 12 months (10,000 KRW); education level, which was categorized as primary school graduation or below, middle or high school graduation, and college graduation or above; and marital status, which was categorized as married, divorced or widowed, and unmarried. Health-related factors included smoking, defined as a lifetime smoking history of 5 packs of cigarettes or more [22]; drinking, defined as alcohol consumption at least once a week; physical activity, measured in MET-h/day using the Physical Activity Scale for Individuals with Physical Disabilities, which is designed to assess physical activity levels in individuals with a physical disability [23]; diabetes and hypertension, determined based on physician diagnosis; and menopausal status.

### 2.7. Data Analysis

Continuous variables were presented as the mean and standard deviation. Categorical variables were presented as frequencies and percentages. Independent-sample *t*-tests and chi-square tests were used to compare continuous and categorical variables according to sex, HGS, and AMI, while binary logistic regression analysis was used to calculate odds ratios (ORs) and 95% confidence intervals (CIs) for hepatic steatosis according to the independent and combined categories of HGS and AMI. All analyses were conducted using SPSS version 29.0 (SPSS Inc., Chicago, IL, USA), and the statistical significance level for hypothesis testing was set at *p* = 0.05.

## 3. Results

### 3.1. Comparison of Variables According to Sex

Table 1 presents the comparison of variables by sex. Men had significantly higher proportions of spinal-cord-injury- and brain-lesion-related disabilities (*p* = 0.028), as well as higher WC (*p* = 0.004), arm muscle mass (*p* < 0.001), AMI (*p* = 0.012), HGS (*p* = 0.003), and smoking rates (*p* < 0.001) but a significantly shorter duration of injury (*p* = 0.009) and lower body fat (*p* = 0.009) than women.

### 3.2. Comparison of Variables According to Relative HGS

Table 2 presents a comparison of variables by relative HGS. The low relative HGS group had significantly higher BMI (*p* = 0.023), WC (*p* < 0.001), HSI score (*p* = 0.009), and FSI score (*p* = 0.010), as well as a significantly higher prevalence of steatosis (HSI, *p* = 0.022; FSI, *p* = 0.040) than the high relative HGS group. Conversely, the AMI relative to body weight (*p* = 0.004) was significantly lower in the low relative HGS group than in the high relative HGS group.

### 3.3. Comparison of Variables According to AMI

Table 3 presents a comparison of variables by AMI. The low-AMI group had significantly higher BMI (*p* = 0.003), percent body fat (*p* = 0.009), WC (*p* = 0.013), HSI score (*p* = 0.006), FSI score (*p* = 0.030), and a higher prevalence of steatosis (HSI, *p* = 0.022; FSI, *p* = 0.003) than the high-AMI group. In contrast, arm muscle mass was significantly lower in the low-AMI group than in the high-AMI group (*p* = 0.003).

### 3.4. ORs for NAFLD Risk According to Relative HGS and AMI

Table 4 presents the calculated ORs for the risk of NAFLD by HGS and AMI. First, for NAFLD by HGS, HSI scores indicated that the odds of high-risk steatosis were significantly higher in the low-HGS group (OR = 3.589, 95% CI = 1.302–9.891, *p* = 0.013) than in the high-HGS group (OR = 1), and the results remained significant even after adjustment for covariates, including socioeconomic status and health-related variables (OR = 3.831, 95% CI = 1.284–11.431, *p* = 0.016). Similarly, FSI scores indicated that the odds of high-risk steatosis were significantly higher in the low-HGS group (OR = 2.710, 95% CI = 1.095–6.711, *p* = 0.031) than in the high-HGS group (OR = 1), and the results remained significant even after adjusting for covariates (OR = 2.747, 95% CI = 1.035–7.286, *p* = 0.003).

For NAFLD by AMI, HSI scores indicated that the odds of high-risk steatosis were significantly higher in the low-AMI group (OR = 2.762, 95% CI = 1.031–7.398, *p* = 0.043) than in the high-AMI group (OR = 1), and the results remained significant even after adjusting for covariates (OR = 3.359, 95% CI = 1.172–9.625, *p* = 0.024). Similarly, FSI scores indicated that the odds of high-risk steatosis were significantly higher in the low-AMI group (OR = 3.493, 95% CI = 1.401–8.711, *p* = 0.007) than in the high-AMI group (OR = 1), and the results remained significant even after adjusting for covariates (OR = 4.714, 95% CI = 1.699–13.078, *p* = 0.003).

### 3.5. ORs for Risk of NAFLD According to Relative HGS and AMI Combined

Table 5 presents the calculated ORs for the risk of NAFLD by combined relative HGS and AMI categories. First, HSI scores indicated that the odds of high-risk steatosis were significantly higher only in the low-HGS + low-AMI group (OR = 6.849, 95% CI = 1.760–26.647, *p* = 0.006) compared with the high-HGS + high-AMI group (OR = 1), and the association remained significant even after adjustment for covariates (OR = 7.352, 95% CI = 1.795–30.104, *p* = 0.006). Similarly, FSI scores also indicated that the odds of high-risk steatosis were significantly higher only in the low-HGS + low-AMI group (OR = 6.957, 95% CI = 1.982–24.421, *p* = 0.002) compared with the high-HGS + high-AMI group (OR = 1), and the association remained significant even after adjustment for covariates (OR = 7.406, 95% CI = 2.007–27.326, *p* = 0.003).

## 4. Discussion

This study examined the independent and combined associations of HGS and AMI with the risk of NAFLD in 85 individuals who use wheelchairs. The findings showed that low HGS and low AMI were both associated with an increased likelihood of NAFLD risk and that low HGS combined with low AMI further strengthened this association.

First, the prevalence of NAFLD risk among individuals who use wheelchairs in this study was 34.1% based on HSI scores and 49.4% based on FSI scores. In an epidemiological study using large-scale statistical data in Korea, the prevalence of NAFLD among individuals without disabilities was estimated to be approximately 21.5% [24]. This indicates that the prevalence of NAFLD in our study population was considerably higher than that reported for individuals without disabilities.

Skeletal muscle is an active metabolic tissue in the body that regulates energy metabolism and insulin sensitivity [25,26]. Impaired energy metabolism and insulin sensitivity contribute to obesity, a risk factor for NAFLD, and the onset of obesity-related diseases [27,28]. In addition, decreased skeletal muscle mass and reduced strength relative to body weight, which reflect muscle status directly and indirectly, have been reported to be independently associated with an increased risk of NAFLD. Consistent with this evidence, three cross-sectional studies that used nationally representative Korean samples [29,30,31] and a cohort study based on a large-scale Chinese population [32] reported that HGS is negatively associated with NAFLD. Moreover, a meta-analysis of 19 observational and cross-sectional studies [14], a cohort study based on a large-scale Korean population [33], and a cross-sectional study using MRI data from the UK Biobank [34] reported that low AMI relative to body weight is significantly associated with an increased risk of NAFLD. Similarly, the present study examined the associations of NAFLD risk with HGS and AMI in individuals who use wheelchairs and found that low HGS and low AMI were significantly associated with the risk of NAFLD. These findings suggest that muscle strength and skeletal muscle mass relative to body weight are significantly associated with the risk of NAFLD in individuals who use wheelchairs. HGS and AMI, in particular, may serve as useful indicators associated with the risk of NAFLD in this population.

Furthermore, muscle strength and skeletal muscle mass are closely correlated but not entirely interdependent. Specifically, loss of muscle strength can only be partially explained by loss of muscle mass [35]. Considering that both loss of muscle strength and skeletal muscle mass are associated with NAFLD, it is plausible that evaluating these two factors together may further strengthen the observed association. Indeed, a recent study by Gan et al. [16] investigated the association between NAFLD and the concurrent decline in muscle strength and mass in adults aged ≥ 18 years and found that the group with both low muscle strength and low muscle mass had a significantly higher risk of NAFLD than groups with either low muscle strength or low muscle mass alone. Similarly, this study examined the association between risk of NAFLD and combined HGS and AMI in individuals who use wheelchairs and found that the group with low HGS combined with low AMI had a significantly higher risk of NAFLD. These findings suggest that maintaining both muscle strength and muscle mass may be relevant to NAFLD-related metabolic health, regardless of disability. These findings also suggest that upper-body exercise strategies that can increase arm muscle mass and arm muscle strength should be emphasized to reduce the risk of NAFLD in individuals who use wheelchairs. This interpretation is supported by a previous study reporting that upper-body exercise for individuals with disabilities who use a wheelchair due to SCI can improve biomarkers related to liver function [36].

Several possible explanations may account for the associations of NAFLD risk with HGS and AMI in individuals who use wheelchairs. First, regular physical activity and exercise are significantly associated with a lower risk of NAFLD [37,38]. Among individuals who use wheelchairs, HGS is an indicator of functional independence [39], and HGS and physical activity are positively correlated [40]. Therefore, the high-HGS group may have been more likely to participate in regular physical activity than the low-HGS group, which may have contributed to a lower risk of NAFLD. Second, the characteristics of physical activity may influence upper-limb muscle development. Individuals who use wheelchairs may experience muscle atrophy because of prolonged disuse of the legs [17], and because their arms are used for movement during daily life, they also show differences in arm muscle development [41]. In other words, people with more developed arm muscles may be more likely to participate in greater levels of physical activity, resulting in a lower risk of NAFLD. Third, muscle loss may increase insulin resistance and chronic inflammation and reduce vitamin D levels and beneficial myokine secretion [13,42]. Such physiological changes due to muscle loss have been reported to be mechanisms linking muscle mass and muscle strength loss to risk of NAFLD [13]. However, the possibility of reverse causality should be considered, as NAFLD itself may contribute to declines in muscle strength and mass. Specifically, NAFLD-related systemic inflammation, characterized by the release of pro-inflammatory cytokines such as TNF-α, can impair muscle protein metabolism [13]. Additionally, the metabolic dysfunction and increased insulin resistance associated with NAFLD may further impair mitochondrial function and overall muscle quality [43]. Therefore, a bidirectional relationship, often termed the ‘liver–muscle axis,’ may exist, in which NAFLD and muscle depletion exacerbate each other.

This study had some limitations. First, because of its cross-sectional design, this study cannot establish causality for the findings. Second, the risk of NAFLD was defined using surrogate indices based on hematological markers (HSI and FSI) rather than imaging-based modalities such as ultrasonography, magnetic resonance imaging, or transient elastography. Although HSI and FSI have been widely used and validated as noninvasive screening tools for NAFLD in previous studies, their validity in individuals who use wheelchairs may be limited because body composition and metabolic characteristics in this population can differ from those of the general population. Therefore, the possibility of misclassifying NAFLD cannot be completely excluded. Third, the terminology used to define fatty liver disease should also be considered when interpreting the findings. The nomenclature has recently evolved from NAFLD to metabolic dysfunction-associated fatty liver disease (MAFLD) and metabolic dysfunction-associated steatotic liver disease (MASLD), reflecting a shift toward a more metabolically oriented disease definition. However, this conceptual transition was not fully established at the time of study design and data analysis. Therefore, the generalizability of the present findings within the MAFLD or MASLD frameworks is limited. Fourth, the study population consisted of 85 individuals who use wheelchairs; thus, the small sample size should be considered. Fifth, important confounding variables, such as insulin resistance, dietary factors, medication use, and detailed physical activity assessment, were not available in this study, which may have influenced the observed associations. Therefore, the results should be interpreted with caution, and future longitudinal studies using more definitive diagnostic methods for NAFLD in larger samples of individuals who use wheelchairs are needed. Despite these limitations, this study is the first to report the independent and combined associations of low muscle strength and low AMI with the risk of NAFLD in individuals who use wheelchairs. The findings may provide useful insight into the reduction in NAFLD risk in this population.

## 5. Conclusions

This study demonstrated that low HGS and low AMI are independently associated with the risk of NAFLD in individuals who use wheelchairs and that combining these two factors further increases the risk of NAFLD. To the best of our knowledge, these findings provide the first evidence of independent and combined associations of muscle strength and muscle mass with risk of NAFLD in individuals who use wheelchairs. The findings also suggest the importance of interventional strategies that can increase both arm muscle strength and arm muscle mass for reducing the risk of NAFLD in this population.

## Figures and Tables

**Table 1:** Characteristics of study participants.

Variables	Total (*n* = 85)	Men (*n* = 59)	Women (*n* = 26)	*p* Value
Age (years)	57.8 ± 9.2	57.0 ± 9.4	59.5 ± 8.7	0.260
Duration of injury (years)	32.4 ± 18.9	28.5 ± 17.0	41.1 ± 20.4	0.009
Type of disability, *n* (%)				0.028
Poliomyelitis	17 (20.0)	7 (11.9)	10 (38.5)	
Spinal cord injury	39 (45.9)	30 (50.8)	9 (34.6)	
Myelitis	3 (3.5)	1 (1.7)	2 (7.7)	
Brain lesions	21 (24.7)	17 (28.8)	4 (15.4)	
Arthritis	5 (5.9)	4 (4.7)	1 (3.8)	
**Body composition parameters**				
Body mass index (kg/m^2^)	26.1 ± 3.9	26.4 ± 3.8	25.2 ± 4.1	0.183
Body fat (%)	41.5 ± 9.3	39.7 ± 9.7	45.4 ± 7.2	0.009
Waist circumference (cm)	91.4 ± 12.1	93.9 ± 11.5	85.8 ± 11.8	0.004
Arm muscle mass (kg)	5.1 ± 1.5	5.5 ± 1.4	4.2 ± 1.3	<0.001
Arm muscle mass index (%)	7.8 ± 1.6	8.0 ± 1.6	7.1 ± 1.6	0.012
**Socioeconomic status**				
Household income (10,000 KRW/month)	200.9 ± 137.7	193.6 ± 128.2	217.5 ± 158.6	0.463
Education, *n* (%)				0.387
Lower than elementary school	8 (9.4)	4 (6.8)	4 (15.4)	
Middle/high school	72 (84.7)	52 (88.1)	20 (76.9)	
College graduation or above	5 (5.9)	3 (5.1)	2 (7.7)	
Marital status, *n* (%)				0.469
Married	54 (63.5)	36 (61.0)	18 (69.2)	
Widowed/divorced/unmarried	31 (36.5)	23 (39.0)	8 (30.8)	
**Health-related factors**				
HGS (%)	47.3 ± 16.4	50.8 ± 16.0	39.3 ± 14.7	0.003
Smoking, *n* (%)	40 (47.1)	38 (64.4)	2 (7.7)	<0.001
Alcohol consumption, *n* (%)	37 (43.5)	27 (45.8)	10 (38.5)	0.532
Physical activity (MET-h/day)	23.1 ± 10.2	21.9 ± 10.0	25.7 ± 10.4	0.113
Hypertension, *n* (%)	44 (51.8)	30 (50.8)	14 (53.8)	0.799
Diabetes, *n* (%)	16 (18.8)	12 (20.3)	4 (15.4)	0.590
Menopause, *n* (%)	21 (24.7)	0 (0.0)	21 (80.8)	<0.001
**Blood markers**				
FBG (mg/dL)	110.8 ± 23.3	112.6 ± 25.6	106.8 ± 16.6	0.295
HDL-C (mg/dL)	52.5 ± 12.5	50.5 ± 10.0	57.2 ± 16.1	0.057
TG (mg/dL)	165.2 ± 82.8	155.8 ± 79.7	186.5 ± 87.4	0.115
AST (IU/L)	34.3 ± 11.2	34.5 ± 12.0	33.8 ± 9.3	0.797
ALT (IU/L)	30.1 ± 14.0	30.3 ± 14.5	29.5 ± 12.9	0.791
GGT (IU/L)	33.5 ± 31.9	32.5 ± 31.4	35.8 ± 33.6	0.669
**NAFLD index**				
HSI score	34.2 ± 5.3	34.0 ± 5.2	34.5 ± 5.7	0.720
FSI score	28.4 ± 21.2	28.9 ± 21.6	27.4 ± 20.7	0.225
**Steatosis prevalence**				
HSI, *n* (%)	29 (34.1)	19 (32.2)	10 (38.5)	0.575
FSI, *n* (%)	42 (49.4)	29 (49.2)	13 (50.0)	0.943

HGS: Hand-grip strength; MET: metabolic equivalent of task; FBG: fasting blood glucose; HDL-C: high-density lipoprotein cholesterol; TG: triglycerides; AST: aspartate aminotransferase; ALT: alanine aminotransferase; GGT: gamma-glutamyl transferase; NAFLD: non-alcoholic fatty liver disease; HSI: hepatic steatosis index; FSI: Framingham steatosis index.

**Table 2:** Descriptive statistics of the measured parameters according to hand-grip strength categories.

Variables	Low HGS (*n* = 41)	High HGS (*n* = 44)	*p* Value
Age (years)	57.4 ± 9.5	58.1 ± 9.0	0.746
Women, *n* (%)	12 (29.3)	14 (31.8)	0.799
Duration of injury (years)	30.2 ± 16.0	34.4 ± 21.3	0.314
Type of disability, *n* (%)			0.339
Poliomyelitis	5 (12.2)	12 (27.3)	
Spinal cord injury	19 (46.3)	20 (45.5)	
Myelitis	2 (4.9)	1 (2.3)	
Brain lesions	13 (31.7)	8 (18.2)	
Arthritis	2 (4.9)	3 (6.8)	
**Body composition parameters**			
Body mass index (kg/m^2^)	27.0 ± 4.3	25.1 ± 3.3	0.023
Body fat (%)	41.2 ± 9.6	41.7 ± 9.2	0.789
Waist circumference (cm)	96.5 ± 11.8	86.7 ± 10.5	<0.001
Arm muscle mass (kg)	5.0 ± 1.1	5.2 ± 1.7	0.581
Arm muscle mass index (%)	7.2 ± 1.1	8.2 ± 1.9	0.004
**Socioeconomic status**			
Household income (10,000 KRW/month)	184.6 ± 123.1	216.0 ± 149.8	0.296
Education, *n* (%)			0.722
Lower than elementary school	3 (7.3)	5 (11.4)	
Middle/high school	35 (85.4)	37 (84.1)	
College graduation or above	3 (7.3)	2 (4.5)	
Marital status, *n* (%)			0.068
Married	22 (53.7)	32 (72.7)	
Widowed/divorced/unmarried	19 (46.3)	12 (27.3)	
**Health-related factors**			
Smoking, *n* (%)	23 (56.1)	17 (38.6)	0.107
Alcohol consumption, *n* (%)	15 (36.6)	22 (50.0)	0.213
Physical activity (MET-h/day)	23.2 ± 13.0	23.0 ± 6.8	0.952
Hypertension, *n* (%)	22 (53.7)	22 (50.0)	0.736
Diabetes, *n* (%)	10 (24.4)	6 (13.6)	0.205
Menopause, *n* (%)	11 (26.8)	10 (22.7)	0.661
**Blood markers**			
FBG (mg/dL)	116.0 ± 27.5	106.1 ± 17.5	0.055
HDL-C (mg/dL)	50.2 ± 9.1	54.7 ± 14.8	0.094
TG (mg/dL)	166.0 ± 74.4	164.5 ± 90.8	0.935
AST (IU/L)	32.9 ± 9.8	35.6 ± 12.3	0.267
ALT (IU/L)	30.9 ± 13.8	29.3 ± 14.2	0.599
GGT (IU/L)	35.2 ± 32.3	31.9 ± 31.8	0.633
**NAFLD index**			
HSI score	35.7 ± 5.6	32.7 ± 4.7	0.009
FSI score	32.8 ± 23.0	24.4 ± 18.8	0.010
**Steatosis prevalence**			
HSI, *n* (%)	19 (46.3)	10 (22.7)	0.022
FSI, *n* (%)	25 (61.0)	17 (38.6)	0.040

HGS: Hand-grip strength; MET: metabolic equivalent task; FBG: fasting blood glucose; HDL-C: high-density lipoprotein cholesterol; TG: triglycerides; AST: aspartate aminotransferase; ALT: alanine aminotransferase; GGT: gamma-glutamyl transferase; NAFLD: non-alcoholic fatty liver disease; HSI: hepatic steatosis index; FSI: Framingham steatosis index.

**Table 3:** Descriptive statistics of the measured parameters according to arm muscle mass index categories.

Variables	Low AMI (*n* = 41)	High AMI (*n* = 44)	*p* Value
Age (years)	59.1 ± 8.0	56.5 ± 10.2	0.205
Women, *n* (%)	12 (29.3)	14 (31.8)	0.799
Duration of injury (years)	35.5 ± 21.0	29.5 ± 16.5	0.151
Type of disability, *n* (%)			0.097
Poliomyelitis	12 (29.3)	5 (11.4)	
Spinal cord injury	18 (43.9)	21 (47.7)	
Myelitis	0 (0.0)	3 (6.8)	
Brain lesions	10 (24.4)	11 (25.0)	
Arthritis	1 (2.4)	4 (9.1)	
**Body composition parameters**			
Body mass index (kg/m^2^)	27.4 ± 3.8	24.8 ± 3.6	0.003
Body fat (%)	44.2 ± 9.2	38.9 ± 8.8	0.009
Waist circumference (cm)	94.8 ± 12.2	88.3 ± 11.4	0.013
Arm muscle mass (kg)	4.7 ± 1.2	5.6 ± 1.6	0.003
**Socioeconomic status**			
Household income (10,000 KRW/month)	201.2 ± 149.7	200.6 ± 127.2	0.983
Education, *n* (%)			0.743
Lower than elementary school	3 (7.3)	5 (11.4)	
Middle/high school	36 (87.8)	36 (81.8)	
College graduation or above	2 (4.9)	3 (6.8)	
Marital status, *n* (%)			0.637
Married	25 (61.0)	29 (65.9)	
Widowed/divorced/unmarried	16 (39.0)	15 (34.1)	
**Health-related factors**			
HGS (%)	44.0 ± 16.2	50.3 ± 16.3	0.079
Smoking, *n* (%)	19 (46.3)	21 (47.7)	0.898
Alcohol consumption, *n* (%)	19 (46.3)	18 (40.9)	0.614
Physical activity (MET-h/day)	23.3 ± 9.1	22.9 ± 11.3	0.851
Hypertension, *n* (%)	23 (56.1)	21 (47.7)	0.440
Diabetes, *n* (%)	7 (17.1)	9 (20.5)	0.690
Menopause, *n* (%)	9 (22.0)	12 (27.3)	0.570
**Blood markers**			
FBG (mg/dL)	111.5 ± 22.3	110.3 ± 24.3	0.815
HDL-C (mg/dL)	52.3 ± 12.3	52.7 ± 12.8	0.874
TG (mg/dL)	171.6 ± 68.9	159.2 ± 94.4	0.495
AST (IU/L)	34.6 ± 10.7	34.0 ± 11.7	0.782
ALT (IU/L)	31.6 ± 13.4	28.6 ± 14.5	0.322
GGT (IU/L)	36.8 ± 32.3	30.5 ± 31.6	0.362
**NAFLD index**			
HSI score	35.8 ± 5.0	32.7 ± 5.3	0.006
FSI score	33.6 ± 21.6	23.7 ± 19.9	0.030
**Steatosis prevalence**			
HSI, *n* (%)	19 (46.3)	10 (22.7)	0.022
FSI, *n* (%)	27 (65.9)	15 (34.1)	0.003

AMI: Arm muscle mass index; HGS: hand-grip strength; MET: metabolic equivalent task; FBG: fasting blood glucose; HDL-C: high-density lipoprotein cholesterol; TG: triglycerides; AST: aspartate aminotransferase; ALT: alanine aminotransferase; GGT: gamma-glutamyl transferase; NAFLD: non-alcoholic fatty liver disease; HSI: hepatic steatosis index; FSI: Framingham steatosis index.

**Table 4:** Odds ratios for hepatic steatosis risk by hand-grip strength and arm muscle mass index.

Variable	Model 1		Model 2	
	OR (95% CI)	*p* Value	OR (95% CI)	*p* Value
**HGS categories**				
**HSI**				
High HGS	1 (reference)		1 (reference)	
Low HGS	3.589 (1.302–9.891)	0.013	3.831 (1.284–11.431)	0.016
**FSI**				
High HGS	1 (reference)		1 (reference)	
Low HGS	2.710 (1.095–6.711)	0.031	2.747 (1.035–7.286)	0.042
**AMI categories**				
**HSI**				
High AMI	1 (reference)		1 (reference)	
Low AMI	2.762 (1.031–7.398)	0.043	3.359 (1.172–9.625)	0.024
**FSI**				
High AMI	1 (reference)		1 (reference)	
Low AMI	3.493 (1.401–8.711)	0.007	4.714 (1.699–13.078)	0.003

OR: Odds ratio; CI: confidence interval; HGS: hand-grip strength; AMI: arm muscle mass index; HSI: hepatic steatosis index; FSI: Framingham steatosis index. Model 1: Adjusted for age and sex. Model 2: Model 1 + body fat, smoking, menopausal status, and disability-related parameters (i.e., duration of injury and type of disability).

**Table 5:** Joint association of hand-grip strength and arm muscle mass index with the risk of hepatic steatosis.

Variable	Model 1		Model 2	
	OR (95% CI)	*p* Value	OR (95% CI)	*p* Value
**HSI**				
High HGS & high AMI	1 (reference)		1 (reference)	
High HGS & low AMI	1.553 (0.346–6.971)	0.566	2.060 (0.404–10.495)	0.384
Low HGS & high AMI	2.089 (0.450–9.711)	0.347	2.406 (0.453–12.793)	0.303
Low HGS & low AMI	6.849 (1.760–26.647)	0.006	7.352 (1.795–30.104)	0.006
**FSI**				
High HGS & high AMI	1 (reference)		1 (reference)	
High HGS & low AMI	1.693 (0.461–6.218)	0.428	2.499 (0.550–11.359)	0.236
Low HGS & high AMI	1.224 (0.322–4.646)	0.767	1.232 (0.282–5.392)	0.781
Low HGS & low AMI	6.957 (1.982–24.421)	0.002	7.406 (2.007–27.326)	0.003

OR: Odds ratio; CI: confidence interval; HGS: hand-grip strength; AMI: arm muscle mass index; NAFLD: non-alcoholic fatty liver disease; HSI: hepatic steatosis index; FSI: Framingham steatosis index. Model 1: Adjusted for age and sex. Model 2: Model 1 + body fat, smoking, menopausal status, and disability-related parameters (i.e., duration of injury and type of disability).

## Data Availability

The data presented in this study are available upon request from the corresponding author due to privacy.

## Abbreviations

The following abbreviations are used in this manuscript:

NAFLD	Non-alcoholic fatty liver disease;
SCI	Spinal cord injury;
HGS	Hand-grip strength;
AMI	Arm muscle mass index;
BMI	Body mass index;
WC	Waist circumference;
FBG	Fasting blood glucose;
HDL-C	High-density lipoprotein cholesterol;
TG	Triglycerides;
AST	Aspartate aminotransferase;
ALT	Alanine aminotransferase;
GGT	Gamma-glutamyl transferase;
HSI	Hepatic steatosis index;
FSI	Framingham steatosis index;
OR	Odds ratio;
CI	Confidence interval.
